# Environmental Concentrations of Copper, Alone or in Mixture With Arsenic, Can Impact River Sediment Microbial Community Structure and Functions

**DOI:** 10.3389/fmicb.2018.01852

**Published:** 2018-08-14

**Authors:** Ayanleh Mahamoud Ahmed, Emilie Lyautey, Chloé Bonnineau, Aymeric Dabrin, Stéphane Pesce

**Affiliations:** ^1^Irstea, UR RiverLy, Centre de Lyon-Villeurbanne, Villeurbanne, France; ^2^CARRTEL, Univ. Savoie Mont Blanc, INRA, Chambéry, France; ^3^Centre de Recherche, Université de Djibouti, Djibouti, Djibouti

**Keywords:** benthic communities, heterotrophic communities, combined effects, metals, microbial ecotoxicology, enzymatic activities, genetic structure

## Abstract

In many aquatic ecosystems, sediments are an essential compartment, which supports high levels of specific and functional biodiversity thus contributing to ecological functioning. Sediments are exposed to inputs from ground or surface waters and from surrounding watershed that can lead to the accumulation of toxic and persistent contaminants potentially harmful for benthic sediment-living communities, including microbial assemblages. As benthic microbial communities play crucial roles in ecological processes such as organic matter recycling and biomass production, we performed a 21-day laboratory channel experiment to assess the structural and functional impact of metals on natural microbial communities chronically exposed to sediments spiked with copper (Cu) and/or arsenic (As) alone or mixed at environmentally relevant concentrations (40 mg kg^-1^ for each metal). Heterotrophic microbial community responses to metals were evaluated both in terms of genetic structure (using ARISA analysis) and functional potential (using exoenzymatic, metabolic and functional genes analyses). Exposure to Cu had rapid marked effects on the structure and most of the functions of the exposed communities. Exposure to As had almost undetectable effects, possibly due to both lack of As bioavailability or toxicity toward the exposed communities. However, when the two metals were combined, certain functional responses suggested a possible interaction between Cu and As toxicity on heterotrophic communities. We also observed temporal dynamics in the functional response of sediment communities to chronic Cu exposure, alone or in mixture, with some functions being resilient and others being impacted throughout the experiment or only after several weeks of exposure. Taken together, these findings reveal that metal contamination of sediment could impact both the genetic structure and the functional potential of chronically exposed microbial communities. Given their functional role in aquatic ecosystems, it poses an ecological risk as it may impact ecosystem functioning.

## Introduction

Sediments are an essential component of aquatic ecosystems, as they provide a habitat for many species and thus host a non-negligible biological diversity (Battin et al., 2001). Within sediments, benthic heterotrophic microbial communities support various ecosystem functions, from organic matter recycling (Schwarz et al., 2007) to pollutant degradation and transformation (Bedard, 2008) and biomass production (Haglund et al., 2003). They are essential for the proper functioning of biogeochemical cycles as well as for ecosystem stability and resilience (Martiny et al., 2013). Sediments are also natural receptors for hydrophobic and persistent pollutants (such as trace metals, polycyclic aromatic hydrocarbons or polychlorinated biphenyls) that can accumulate over time (Bombardier, 2007). Physicochemical disturbances (such as changes in pH or redox potential), as well as mechanical (e.g., dredging) or biological (e.g., bioturbation) changes in environmental conditions can induce a release of contaminants into interstitial water, thus increasing their bioavailability and their ecotoxicological risks (Faupel et al., 2012). Sediments will then become a source of pollutants for the hosted benthic communities (Fernandez-Calvino et al., 2008; Burga Pérez et al., 2012), which can be directly exposed and structurally and functionally impacted by the contaminant compounds.

Trace metals, which include metals and metalloids, are ubiquitous and persistent in the environment. They are found in all aquatic compartments due to natural (e.g., physical and chemical alteration of rocks) and anthropogenic (e.g., industry, agriculture, etc.) inputs. Copper (Cu) and arsenic (As) are two of the most common metals present in sediments (Serra et al., 2009; Rigaud, 2011) and both Cu- and As-contaminated areas are constantly increasing due, among other factors, to mining activities or to their use as pesticides in both conventional and organic (for Cu) agriculture (Achour-Rokbani et al., 2010; Bereswill et al., 2013). Copper is an essential trace element serving as a cofactor in many enzyme pathways that catalyze a wide variety of biological functions in all organisms (e.g., Ladomersky and Petris, 2015; Adams et al., 2016) and contribute to normal ecosystem functioning. In the environment, As is mainly present in inorganic forms and in different states of oxidation, but primarily as arsenite (oxidation state +3) and arsenate (oxidation state +5). Nevertheless, sediments also contain other forms, such as arsenobetaine generally derived from biological activities (Hettiarachchi et al., 2017).

Excessive doses of both Cu and As can induce toxic effects on living organisms such as heterotrophic microorganisms. There is strong evidence that increasing concentrations of Cu and As can impact bacterial diversity (e.g., Turpeinen et al., 2004; Dell’Amico et al., 2008; Tlili et al., 2010, 2011), bacterial growth (Poirel et al., 2013), and several microbial heterotrophic functions, including glucose consumption (Poirel et al., 2013), extracellular enzymatic activities such as beta-glucosidase activity (Lambert et al., 2012) and substrate-induced respiration (Tlili et al., 2011) in various contaminated compartments such as soils and surface freshwaters. However, there is a big gap in research on the effects of Cu and As (and metals in general) on freshwater sediment microbial heterotrophic communities, especially studies on environmentally relevant concentrations and potential Cu–As interactions.

Studies in marine sediment have demonstrated that about 50 mg Cu kg^-1^ can decrease the ability of bacteria to use carbon sources (Gillan, 2004) and that concentrations up to 33 mg Cu kg^-1^ can reduce the density of heterotrophic bacteria and modify the community structure (Zhao et al., 2014). In Xiangjiang River sediment, Jie et al. (2016) observed an increase in the abundance of metal resistance genes in three sites polluted by metals including Cu (392–570 mg kg^-1^) and As (177–2480 mg kg^-1^). Flemming and Trevors (1988) showed in a microcosm study that increasing concentrations of Cu, from 100 to 5000 mg Cu kg^-1^, caused a progressive decrease in freshwater sediment pH and biological nitrous oxide (N_2_O) reduction. Capone et al. (1983) also observed a significant but transient inhibition of both respiration and methanogenesis in salt marsh sediment contaminated by 1000 mg Cu kg^-1^. Such inhibition can lead to significant inhibition of CO_2_ and CH_4_ fluxes as previously reported in soil submitted to Cu treatment (Babich and Stotzky, 1985). Finally, it appears from both *in situ* (Roane and Kellogg, 1996) and laboratory studies (Knight et al., 1997) that bacterial biomass, diversity and metabolic activity in contaminated soils can be correlated to metals concentrations.

In sediment, microorganisms play an important role in biogeochemical cycles of metals, including As, which can be converted to various chemical species differing in their solubility, mobility, bioavailability and toxicity (Silver and Phung, 2005). Using the Microtox bioassay, Fulladosa et al. (2005) showed that acute toxicity of As(V) on *Vibrio fischeri* increased with pH (from 5 to 8) while As(III) toxicity was stable within pH range 6 to 8. Arsenic can inhibit many enzymes that play roles in cellular energy pathways and DNA replication and repair and the formation of As(III)-sulfur bonds can increase its toxicity toward enzymatic activities such as glutathione reductase or thioredoxin reductase (Chang et al., 2003). It can also substitute phosphate in energy compounds such as ATP (Ratnaike, 2003).

Given the frequent occurrence of both Cu and As in freshwater sediments, as well as the crucial ecological role of heterotrophic microorganisms in this compartment, there is a need to improve the environmental risk assessment of these metals in aquatic ecosystems by developing ecotoxicological approaches that address their potential effects, at environmental concentrations, on the structure and functions of benthic microbial communities. In a broader perspective, this type of research is needed to better understand and predict the ecological effects of contaminants in freshwater sediments by better taking into account benthic communities, including microorganisms, in sediment ecotoxicology (Pesce et al., 2018).

Accordingly, the aim of this study was to evaluate the structural and functional effects of chronic exposure to environmental particulate concentrations of Cu and As on natural river sediment microbial communities. The tested nominal concentration was 40 mg kg^-1^ dry weight (dw) for each metal. This concentration is representative of high (i.e., third quartile) and very high (i.e., ninth decile) sediment contamination levels of Cu and As, respectively, in French aquatic ecosystems (INERIS, 2010; DREAL-REMIPP, 2013). To address the potential ecotoxicological interactions between the two metals, we also assessed the effects of Cu and As in mixtures using the same individual concentrations. To this end, we performed a 21-day (d) experiment in laboratory channel microcosms containing natural river sediment initially spiked or not with Cu and/or As. The response of benthic microbial communities to chronic metal exposure was evaluated throughout the experiment, both in terms of genetic structure (using ARISA analyses) and functional potential (using exoenzymatic, metabolic and functional genes analyses).

## Materials and Methods

### Experimental Design

Natural sediment and associated communities were sampled on June 2016 on the Ain River (at Pont de Chazey, 45°54′38.80 N – 5°14′11.18 E), a tributary of the Rhône River (France). About 100 kg of wet surface sediment (0–3 cm) was collected using an Ekman grab and brought back to the laboratory for the experiment after sieving at 2 mm. After sediment homogenization, a subsample was retrieved for the initial characterization of the sediment (particle size distribution by laser diffraction, water content by drying overnight at 105°C, particulate organic carbon and particulate trace metal concentrations). Organic matter was measured by loss on ignition (Heiri et al., 2001).

Four experimental treatments were designed: (i) reference condition (REF) without Cu and As; (ii) Cu-contaminated condition (Cu) with 40 mg Cu kg^-1^ dw; (iii) As-contaminated condition (As) with 40 mg As kg^-1^ dw; and (iv) mixture (MIX) condition with a combined contamination of Cu and As at 40 mg kg^-1^ dw each. The sediment spiking mixture was prepared in 15-L mixing tanks taking into account water content (24%) and sediment density (1.24). The spiking procedure used a 60/40 ratio (vol/vol: sediment/solution) to allow proper homogenization between sediment and the spiked solution which was made with a mixture of groundwater (⅓) and demineralized water (⅔) contaminated with or without (REF treatment) CuSO_4_-5 H_2_O (CAS No. 7758-99-8) and/or AsNaO_2_ (CAS No. 7784-46-5) at 1 g L^-1^. After the sediment spiking procedure, sediment was stirred for 6 h and decanted overnight before distribution into glass indoor channels (L × W × H = 83 cm × 11 cm × 10 cm). Each channel contained 3.5 kg of sediment and was filled with 6 L of recirculating water (4 L demineralized water + 2 L groundwater). Each treatment was replicated in three independent channels. Each channel was connected to a 20-L glass tank (i.e., one independent tank per channel) through an aquarium pump (NEWA MJ 750) for water recirculation at a flow of about 1.5 L min^-1^. Sediment and associated microbial communities were exposed for 21 days in these laboratory channels.

Sediments were sampled for metal analysis at the beginning (Day 0, d0) and the end (Day 21, d21) of the experiment, while sediments for microbial analysis and overlaying waters were sampled at d0 and after 7 (d7), 14 (d14), and 21 (d21) days of exposure. For the microbial structure analysis, the sediment samples were stored at -20°C immediately after the sampling procedure at the different sampling times. Functional analyses were performed within a few hours after sampling, except for the initial sampling time (d0) when sediments were stored for two days at 6°C before activity measurements. Water pH, dissolved oxygen, conductivity and temperature were also measured at each sampling time using portable meters (WTW, Germany).

### Water and Sediment Chemical Analyses

At each sampling time, water samples (100 mL) were collected from each channel into glass vials to determine inorganic nutrient (ammonia, nitrate, nitrite and phosphate) and total organic carbon (TOC) concentrations using French and ISO-standard procedures (i.e., NFT 90-015-2 NF EN 26777, NF EN ISO 10304, NF EN ISO 6878, and NF EN 1484, respectively). In addition, for metals analysis, 40 mL was collected into 50 mL polypropylene (PP) tubes (Sarstedt) after filtration with a PP syringe through 0.45-μm PVDF filters. Filtered samples were then acidified with Suprapur nitric acid 65% (0.5% v/v) and stored in the dark at 4°C before analysis of Cu and As concentrations. Cu and As concentrations in filtered samples were measured by inductively-coupled plasma mass spectrometry (ICP-MS, X7, Thermo Electron Series II) according to French standard NF EN ISO 17294.2. Limits of quantification were 0.05 μg L^-1^ for Cu and 0.02 μg L^-1^ for As.

Copper and arsenic concentration in the sediments were measured from composite samples (30 mL) collected in each mixing tank used for the spiking procedure (d0) and in each channel (d21) and stored in PP tubes. After drying at 40°C and grinding with a ball mill (agate beads and bowl), samples were kept dry in a desiccator. Approximately 300 mg of crushed and homogenized sediments were mineralized with aqua regia (2 mL HNO_3_ and 6 mL HCl SUPRAPUR quality) in a microwave digester (MARS-6 from CEM) in teflon reactors (180°C for 15 min). The mineralized sample was made up with ultrapure water (Veolia) and diluted to obtain a volume of 50 mL. Cu and As concentrations in sediment extracts were measured by inductively-coupled plasma mass spectrometry (ICP-OES 720 ES, series 700) according to French standard NF EN ISO 11885. Water content was measured in order to correct the results for potential moisture. The limits of quantification were 0.66 mg kg^-1^ for Cu and 1.6 mg kg^-1^ for As. REF sediment samples at d0 were mineralized and analyzed in triplicates, allowing to assess the relative standard deviation (RSD) of As and Cu concentrations, which were below 11 and 19%, respectively.

### Analysis of Microbial Community Function

#### Aerobic Respiration and Denitrification Metabolic Measurements

The slurry technique (Furutani et al., 1984) was used to measure aerobic respiration and denitrification rates following the protocol previously described by Foulquier et al. (2013). Briefly, 10 g of wet sediment were immersed in 10 mL of distilled water under aerobic conditions (aerobic respiration) or 10 mL of a KNO_3_ (2.16 g L^-1^) solution under anaerobic conditions (denitrification) in 150-mL glass flasks. Incubation flasks assigned to denitrification measurements were purged three times with He to achieve anaerobiosis, and internal pressure was then adjusted to atmosphere. Fifteen mL of acetylene (C_2_H_2_, 10% v/v final volume) was added to inhibit N_2_O reductases. All samples were incubated at 20°C in the dark under gentle shaking. After 2 h and 5 h, headspace gasses were sampled and analyzed by gas chromatography on an MTI 200 microcatharometer (MTI Analytical Instruments). Aerobic respiration and denitrification were expressed as ng of CO_2_ or N_2_O per g of sediment dw^-1^ h^-1^, respectively.

#### Exoenzymatic Activities Measurements

Potential activities of β-glucosidase (β-glu, EC 3.2.1.21), phosphatase (Pase, EC 3.1.3.1) and leucine aminopeptidase (LAP, EC 3.4.11.1) were measured on wet sediments (1.2 g) by fluorimetry according to the methods described by Foulquier et al. (2013). The substrate-fluorogenic sets for the three enzymatic activities were: 4-methylumbelliferyl-β-D-glucopyranoside (MUF-Glu, CAS No. 18997-57-4) for β-glu, L-leucine-7-amido-4-methylcoumarin hydrochloride (MCU-Leu, CAS No. 62480-44-8) for LAP and 4-methylumbelliferyl phosphatase (MUF-P, CAS No. 3368-04-5) for Pase. The optimal substrate concentrations (i.e., 1000 μM for β-glu, 1333 μM for LAP, and 500 μM for Pase) were determined prior to the experiment. After a 30-min incubation at 20°C in the dark under shaking conditions, activities were stopped with 0.3 mL of glycine buffer (pH 10.4, glycine 0.05 M and NH_4_OH 0.2 M) before centrifugation (at 5000 × *g* for 5 min). Fluorescence was measured using a microplate reader (Synergy HT BioTek Instruments) with excitation wavelength set to 360 nm and emission wavelength set to 460 nm. Enzymatic activity was quantified using standard curves of the reference compounds: MUF (for β-glu and Pase, Sigma M1381 CAS No. 90-33-5) and AMC (for LAP; Sigma A9891, CAS No. 26093-31-2). Results were expressed as nmol of hydrolyzed compound per g of sediment dw^-1^ h^-1^.

#### Analysis of Microbial Community Structure

Microbial sediment DNA was extracted from 0.5 g of wet sediment (stored at -20°C) using a NucleoSpin Soil Kit (Macherey-Nagel EURL) following the manufacturer’s instructions, and using SL1 lysis buffer and additive Enhancer SX buffer. The extracted DNA was quantified fluorometrically after staining with bisBenzimide (DNA Quantitation Kit, Fluorescence Assay, Sigma-Aldrich) using a Plate Chameleon^TM^ fluorometer (Hidex; excitation: 340 nm, emission: 460 nm).

Total and functional bacterial community sizes were estimated using real-time quantitative PCR (qPCR) assays targeting 16S rRNA gene (total bacteria), and *nir*S, *nir*K, and *nos*Z (clades I and II) genes involved in denitrification activity, according to protocols described in **Supplementary Table 1** (Henry et al., 2004, 2006; López-Gutiérrez et al., 2004; Throbäck et al., 2004; Kandeler et al., 2006; Jones et al., 2013). Reactions contained 1 × Master Mix, 0.3 mg mL^-1^ BSA (Sigma-Aldrich), each primer, and 1 μL of template DNA. Standard ranges were plotted from a 10-fold serial dilution of a plasmid solution containing the gene of interest.

Bacterial community structure was assessed using automated ribosomal intergenic spacer analysis (ARISA) based on the length polymorphism of intergenic spacer (ITS) sequences between 16S rRNA and 23S rRNA genes. This method is based on a size polymorphism of the ITS which makes it possible to discriminate organisms by their molecular fingerprint known as Operational Taxonomic Unit (OTU). Amplification was carried out using 5′-6-carboxyfluorescein (FAM)-labeled-S-D-Bact-1522-B-S-20 and L-D-Bact-132-a-A-1 primers (Normand et al., 1996) with 1 ng of template DNA and using a previously described protocol (Lyautey et al., 2011). Amplification products were quantified and purified as described in Billard et al. (2015) before being separated on ABI 3730xl DNA Analyzer (BIOfidal DTAMB, IFR 41, Université Lyon 1) using the internal size standard Dye 5 ladder, 50–1000 bp (Gel company).

#### Statistical Analysis

After confirming normality of the residuals (Shapiro–Wilk test; Royston, 1982) and data homoscedasticity (Fligner-Killeen test; Conover et al., 1981) for all parameters, significant differences between conditions were identified by analysis of variance (ANOVA) and further analyzed with a *post hoc* Tukey test, using R software (version 3.4.3, R Core Team, 2018). At day 21, a 2-factor ANOVA was performed to identify potential interactions between Cu and As on biological parameters. Results were considered significant at *p <* 0.05. Raw ARISA electropherograms were analyzed using Applied BioSystems Peak Scanner software, following the protocol described elsewhere (Billard et al., 2015). The data analyzed was transformed to an abundance matrix, and Bray-Curtis similarities (BC) between samples were calculated. Distances between samples were represented by non-multidimensional scaling (nMDS, Clarke and Warwick, 2001) with R software (Vegan package). A stress value below 0.20 indicates a good representation of the BC distances between samples. Analysis Of Similarity (ANOSIM, R software, Vegan package) was used to test for significant differences between user-defined (*a priori*) groups. ANOSIM was computed from the BC similarity matrix, and a random permutation test (10,000 permutations) was applied. The test was considered significant at *P* < 0.05 after application of the Bonferroni correction (R software, stat package). SIMPER (Similarity Percentage) analysis carried out using PAST software (Hammer et al., 2001) was used to identify the OTUs that contributed to the discrimination of communities from different treatments. The BC measure is implicit to SIMPER.

## Results

### Channel Water and Sediment Chemistry

In the channels, at the water-sediment interface, temperature was 19.2 ± 0.2°C, pH was 8.2 ± 0.9, oxygen concentration was 8.2 ± 1.9 mg L^-1^, oxygen saturation was 90.0 ± 5.1%, and conductivity was 230.0 ± 8.8 μS cm^-1^ (*n* = 48, data not shown), without significant difference between treatments throughout the experiment. Sediment particle-size classes were distributed as follows: 70% of 250–300 μm (fine-to-medium sand), 2% [80–250 μm] (fine sand), 16% [70–80 μm] (very fine sand), 2% [10–20 μm] (medium silt), 8% silt and <1% clay, corresponding to a relatively coarse sediment. There were no between-channel differences for organic matter (3.0 ± 2.1%) and water (24.0 ± 0.9%) contents.

At d0, As and Cu concentrations in the REF treatment were close to 3 mg As kg^-1^ and 2 mg Cu kg^-1^ (**Table 1**). As concentrations in the As and MIX treatments were quite similar and close to 31 mg kg^-1^. Cu concentrations were substantially higher and close to 56 mg kg^-1^ in both Cu and MIX treatments. The difference between Cu and As concentrations at d0 was explained by their contrasted behavior since solubility of Cu is more limited than for As (Smedley and Kinniburgh, 2002). During the 21-day experiment, As and Cu concentrations, respectively, decreased from 16 to 21% and from 13 to 23% in sediments according to treatments (**Table 1**).

**Table 1:** Concentrations of Cu and As in river sediment (mg kg^-1^ dw) at d0 (data from spike mixing tanks) and d21 (data from the three replicated channels: average ±*SD*).

	As (mg kg^-1^ dw)		Cu (mg kg^-1^ dw)	
	d0	d21	d0	d21
REF	2.89	3.12 ± 0.10	1.81	1.30 ± 0.16
As	31.30	26.20 ± 1.2	1.75	2.60 ± 1.20
Cu	3.24	2.96 ± 0.13	56.60	43.60 ± 2.60
MIX	31.20	24.66 ± 0.45	55.10	47.80 ± 2.50

*The four treatments are: sediment without contamination (REF), sediment contaminated with Cu (Cu), sediment contaminated with As (As), and sediment contaminated both with As and Cu (MIX).*

In the overlying and circulating water of the REF channels, dissolved Cu and As concentrations were low (Cu < 2.3 μg L^-1^ and As < 1.4 μg L^-1^) and relatively stable throughout the study. An important release of Cu and a much higher one of As from sediment to water was recorded as illustrated by the dissolved concentrations of Cu (38.9 to 68.9 μg L^-1^) and As (759 to 2829 μg L^-1^) recorded in contaminated channels (**Supplementary Table 2**).

### Aerobic Respiration and Denitrification Activities

**FIGURE 1:**
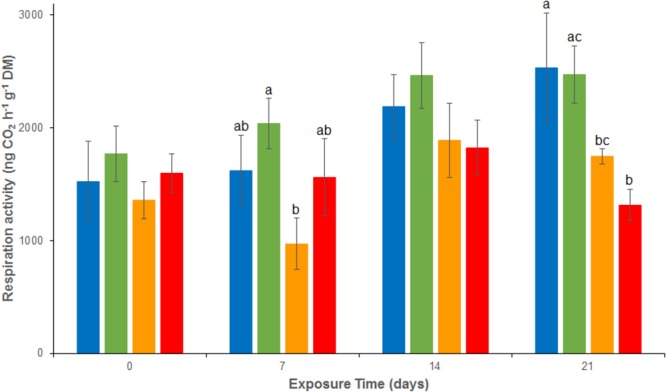
Time–course of average (± standard deviation) microbial respiration activity in uncontaminated sediments (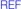) and sediments contaminated with arsenic (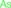), copper (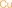) or As plus Cu mixture (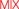). For each sampling time, different letters indicate significant differences between treatments (*P* < 0.05, Tukey test).

Over the experiment, aerobic respiration activities ranged from 972 ± 65 to 2532 ± 485 ng CO_2_ g^-1^ dw h^-1^ (**Figure 1**). No significant difference was observed between REF and As treatments from d0 to d21 or between REF and Cu and between REF and MIX treatments from d0 to d14. At d21, a significant inhibition of respiration was observed in the sediment contaminated by Cu, alone or in mixture (Cu and MIX), compared to the REF and As channels. Indeed, aerobic respiration activities were 1748.0 ± 65.6 and 1316.0 ± 136.1 ng CO_2_g^-1^ dw h^-1^ in Cu and Mix conditions, respectively, while mean values were close to 2500 ng CO_2_g^-1^ dw h^-1^ in both REF and As treatments. No significant interaction between Cu and As on respiration was observed at day 21 (*p* > 0.05 for the interaction term of the 2-factors ANOVA).

**FIGURE 2:**
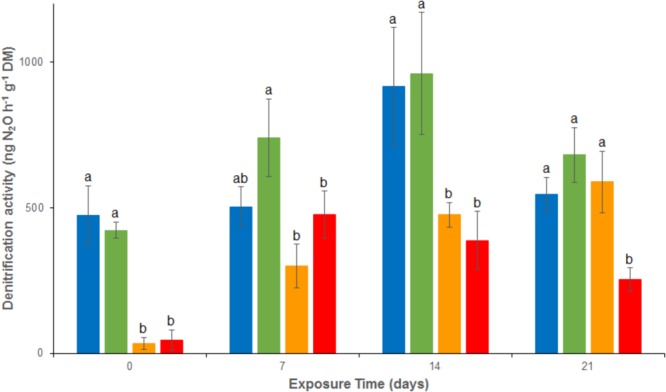
Time–course of average (± standard deviation) microbial denitrification activity in uncontaminated sediments (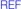) and sediments contaminated with arsenic (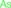), copper (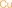) or As plus Cu mixture (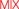). For each sampling time, different letters indicate significant differences between treatments (*P* < 0.05, Tukey test).

Denitrification activity showed no significant difference between REF and As treatment between d0 and d21, with relatively stable activities ranging from 423 ± 28 to 681 ± 93 ng N_2_O g^-1^ dw h^-1^ throughout the experiment (**Figure 2**). However, denitrification was rapidly and significantly inhibited under Cu exposure, alone or in mixture (Cu and MIX: denitrification about 45 ng N_2_O g^-1^ dw h^-1^ at d0). This inhibition lasted throughout the experiment in the MIX channels, with activities ranging from 45 ± 25 to 476 ± 81 ng N_2_O g^-1^ dw h^-1^, whereas the Cu channels showed a recovery at d21 (588 ± 105 ng N_2_O g^-1^ dw h^-1^; no significant difference to REF and As treatments). The difference observed between the exposure to Cu and to MIX suggests an interaction effect between Cu and As which was confirmed by the 2-factor ANOVA (*p* < 0.05 for the interaction term).

### Exo-Enzymatic Activities

**FIGURE 3:**
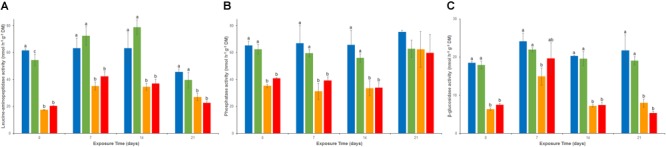
Time–course of average (± standard deviation) microbial leucine-aminopeptidase **(A)**, phosphatase **(B)**, and beta-glucosidase **(C)** activities in uncontaminated sediments (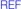) and sediments contaminated with arsenic (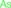), copper (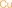), or As plus Cu mixture (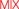). For each sampling time, different letters indicate significant differences between treatments (*P* < 0.05, Tukey test).

Except for a transient and limited inhibition of LAP activity in the As treatment at d0, there were no significant differences throughout the experiment between REF and As for LAP (activity from 39 to about 79 nmol g^-1^ dw h^-1^, **Figure 3A**), Pase (activity from 55 to 75 nmol g^-1^ dw h^-1^, **Figure 3B**) and β-glu (activity from 19 to 23 nmol g^-1^ dw h^-1^, **Figure 3C**). However, all three enzymatic activities were significantly inhibited from d0 in both Cu and MIX channels. This inhibition lasted throughout the 21-day experiment for LAP (**Figure 3A**) and β-glu (except at d7 in the MIX channels; **Figure 3C**) whereas Pase activity showed recovery at d21 in both Cu and MIX channels (**Figure 3B**). No significant interaction between Cu and As on any of the exoenzymes tested was observed at day 21 (*p* > 0.05 for the interaction term of the 2-factors ANOVA).

### Total and Functional Community Abundances

**FIGURE 4:**
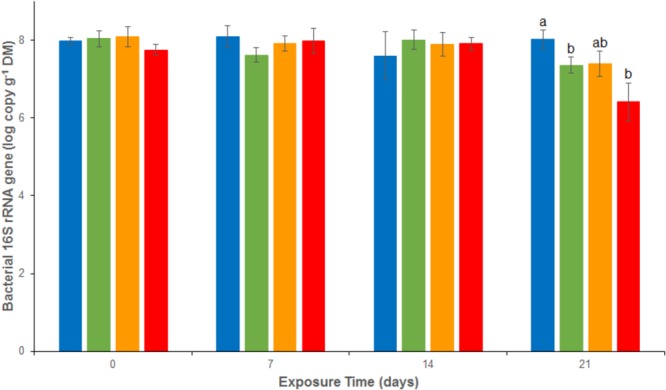
Abundance of *16S* rRNA genes analyzed by quantitative PCR in uncontaminated sediments (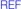) and sediments contaminated with arsenic (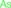), copper (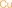), or As plus Cu mixture (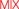). For each sampling time, different letters indicate significant differences between treatments (*P* < 0.05, Tukey test).

**FIGURE 5:**
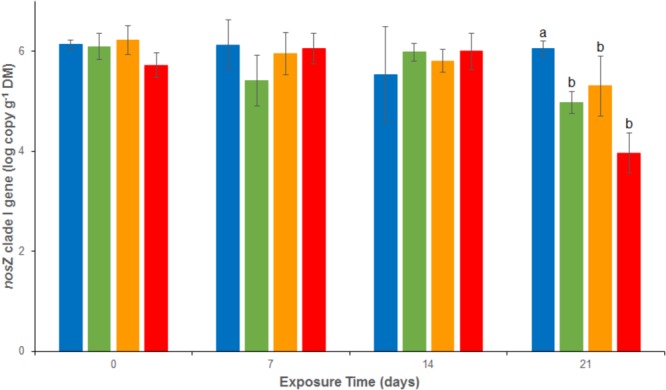
Abundance of *nosZ* clade I gene analyzed by quantitative PCR in uncontaminated sediments (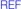) and sediments contaminated with arsenic (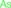), copper (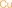), or As plus Cu mixture (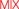). For each sampling time, different letters indicate significant differences between treatments (*P* < 0.05, Tukey test).

Bacterial abundances assessed as 16S rRNA gene abundance were relatively stable and close to 1.2 10^8^ copies g^-1^ dw in the REF treatment throughout the experiment (**Figure 4**). There were no significant differences between the 4 treatments from d0 to d14. At d21, a significant decrease was observed in both As (2.2 10^7^ ± 1.3 10^7^ copies g^-1^ dw), Cu (3.1 10^7^ ± 2.1 10^7^ copies g^-1^ dw) and MIX (0.4 10^7^ ± 0.4 10^7^ copies g^-1^ dw) treatments. The same trend was observed for *nos*Z clade I (**Figure 5**), with stable abundances in the REF treatment from d0 to d21 (average abundance of 1.2 10^6^ ± 9.0 10^5^ copies g^-1^ dw), no significant differences among treatments from d0 to d14, but a significant decrease at d21 in As (1.1 10^5^ ± 0.5 10^5^ copies g^-1^ dw), Cu (3.4 10^5^ ± 2.8 10^5^ copies g^-1^ dw), and MIX (1.3 10^4^ ± 1.1 10^4^ copies g^-1^ dw) treatments. Differences between treatments were also observed in *nirK* and *nirS* abundance (**Supplementary Figure 1**). In particular, after 21 days of exposure *nirK* abundance was higher in sediments exposed to metals than in REF, this increase was significant for sediments exposed to As only. In addition, at the end of the experiment, *nirS* were less abundant in MIX than in Cu. The differences observed in *nosZ* I, *nirS* and *nirK* abundances between exposure to metal alone (Cu or As) and exposure to MIX suggest an interaction effect which was confirmed, for the three genes, by the 2-factor ANOVA (*p* < 0.05 for the interaction term).

For *nosZ* clade II, there were no differences between treatments at any sampling time-points (**Supplementary Figure 2**). Average abundances for the four treatments and the four sampling dates were 8.4 10^7^ ± 0.5 10^7^ copies g^-1^ dw for *nos*Z clade II.

### Bacterial Community Structure

**FIGURE 6:**
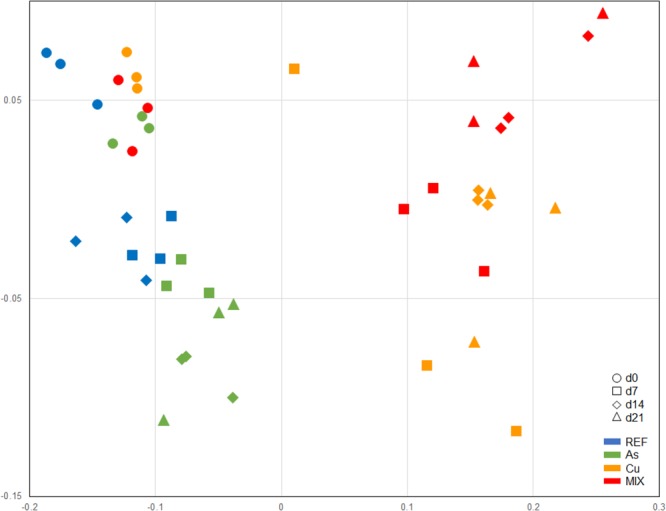
nMDS representation of distances between bacterial communities (stress = 0.137) based on ARISA Bray-Curtis similarity analysis. Symbols are as follows: d0(○), d7(□), d14(♢), and d21(Δ). Treatments are color-coded as follows: 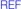, 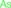, 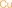, and 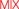.

A total of 474 distinct OTUs was recovered from the ARISA analysis for the whole set of samples (data not shown). The nMDS representation of bacterial community structure (**Figure 6**) and the ANOSIM analysis allowed to significantly discriminate three different groups of samples: (i) the first one clustering all the d0 samples from the 4 treatments, (ii) the second one clustering d7, d14, and d21 samples from both the REF and As treatments, and (iii) the third one clustering d7, d14, and d21 samples from both the Cu and MIX treatments. Half of the calculated dissimilarity between the REF/As group and the Cu/MIX group (from d7 to d21) can be attributed to only 26 OTUs. Among these 26 OTUs, nine were under-represented in the REF/As group (average relative abundances ranging between 0.01 and 2.6%) compared to the Cu/MIX group (1.6–25.7%), contributing to 29% of the dissimilarity. In contrast, the other 17 OTUs were over-represented in the REF/As group with average relative abundances comprised between 1.3 and 4.6% whereas they were below 0.97% in the Cu/MIX group (21% contribution to dissimilarity) (**Figure 6**).

## Discussion

The experimental approach reported here was designed to assess the ecotoxicological effects of As and Cu alone and in mixture on natural sediment microbial community structure and functions, and highlighted the strong effect of Cu on microbial communities.

### Microbial Response to As Exposure

The 21-day exposure to As alone at environmental concentrations close to 25–30 mg kg^-1^ had undetectable or very limited effects on the measured parameters, with only a transient and limited inhibition of LAP activity at the very beginning of the experiment (i.e., after the post-spiking 6-h stirring procedure, the overnight decantation and the 2-day storage before the first activity measurement), a decrease in bacterial abundance and an increase in *nirK* gene abundance and a decrease in *nos*Z clade I gene abundance (without any decrease in denitrification activity as estimated by gas chromatography) at the last sampling date.

Several hypotheses can be proposed to explain the absence of more significant effects. First, this could be due to a low toxicity potential of As to heterotrophic microorganisms at the tested concentrations, which is representative of the very highest (i.e., <10%) As sediment contamination levels recorded in French aquatic ecosystems (INERIS, 2010). Toxicant effects on microbial communities depend not only on its intrinsic toxicity but also on the community’s capacity to resist and tolerate this toxicity. Some microorganisms have thus developed mechanisms of resistance toward trace metal elements. Using the *ars* operon system, bacteria and archaea are able to resist inorganic forms of As by reducing As(V) to As(III) which is then exported out of the cell (Tisa and Rosen, 1990; Rosen, 1999). As it was previously demonstrated in soils that As-resistant microorganisms can be abundant even in As-free environments (Jackson et al., 2005), microbial sediment communities studied here might have been naturally resistant to As. Based on the pollution-induced community tolerance concept first introduced by Blanck et al. (1988), microbial sediment communities might also have acquired resistance to As due to chronic exposure to the low As concentration (i.e., about 3 mg kg^-1^ dw) observed in the river sediment from the sampling location. Indeed, Tuulaikhuu et al., 2015 demonstrated that As concentrations close to 0.6 mg kg^-1^ dw could cause a high mortality in sediment bacterial communities and a significant decrease in phosphatase activity per sediment surface area after a 60-day exposure period. Such results thus suggest that low concentrations of As could be sufficient to induce tolerance changes in bacterial communities due to the elimination of the most sensitive species. Quantification of resistant bacteria using real-time PCR quantification of resistances genes (Poirel et al., 2013) would be a relevant strategy to test this hypothesis.

A low As availability could also explained the limited effect of As observed in the present study. Indeed, As speciation and/or As adsorption to iron oxide (Leiva et al., 2014) might have influenced As availability and then microbial exposure. Indeed, As adsorption to iron oxides also exerts a strong influence on its mobility, being a major natural process for its removal and sequestration, and thus decreasing its bioavailability (Wang and Mulligan, 2006; Drahota et al., 2012; Zambrano, 2012). In the present study, sediment carbonate content was 49% and iron concentration was close to 12.9 g kg^-1^ dw. Since it is generally admitted that sediment iron oxide concentration is inversely related to carbonate content, these values indicate that As adsorption to iron oxide was probably very limited in our case (Lombi et al., 1999; Thirunavukkarasu et al., 2003), and the very well-oxygenated environment in the artificial channels was favorable for the As(V) form.

In addition, arsenic toxicity may also be influenced by its speciation: As(III) being generally more toxic and mobile than As(V), its presence usually causes higher environmental health risks and concerns (Mondal et al., 2006). Further analyses are needed to identify As species in contaminated sediment using a sequential extraction (Keon et al., 2001) and thus to assess any possible changes in As speciation that could explain the limited effect of this metal in our experiment.

### Microbial Response to Cu Exposure, Alone and in Mixture With As

The 21-day exposure to Cu alone at environmental concentrations close to 45–55 mg kg^-1^ strongly affected most of the microbial parameters investigated. Despite a limited effect of Cu on bacterial abundance, which was only detectable at d21 based on quantification of 16S rRNA genes, bacterial community structure showed a clear shift during the first week of exposure that continued until the end of the experiment.

Chronic effects of Cu exposure on bacterial community structure have already been shown in various environmental compartments including soils (Wang et al., 2007), periphytic biofilms (Lambert et al., 2012) and sediments (Zhao et al., 2014). Comparing the structural response of marine bacterial communities collected from the water column, sediments, rock surfaces, and the green seaweed *Ulva compressa*, Moran et al. (2008) demonstrated that sediment communities appeared to be the most vulnerable to Cu exposure, based on terminal restriction fragment length polymorphism analysis.

Besides impacting the bacterial community structure, Cu exposure led to a rapid inhibition of several heterotrophic functions. These functional negative effects were sometimes recorded from the very beginning of the experiment (i.e., d0) while no structural change was observed in bacterial community at this sampling date. Two (non-exclusive) hypotheses could explain this result. Firstly, effects on bacterial communities may have occurred immediately after sediment spiking (i.e., during the 6-h stirring procedure and the overnight decantation) thus possibly precluding the detection of significant shifts on bacterial community structure using DNA genotyping within such a short time scale. Secondly, it can’t be excluded that the initial functional effects mainly occurred during the 2-day storage period used before activity measurements of the d0-samples. In this case, such effects could not be related to structural changes since d0-samples for molecular analysis were immediately frozen after sampling and did not experience this storage period. Indeed, based on gas chromatography analysis, we observed an inhibition of denitrification from the beginning of the exposure despite a lack of significant effect on abundance of *nosZ* clade I gene from d0 to d14. The three enzymatic activities LAP, Pase and β-glu activities were also significantly reduced from d0. The negative effects of metal contamination on various enzyme activities have been recognized in different kinds of soils (Renella et al., 2003; Hinojosa et al., 2004; Wyszkowska et al., 2010). After a 25- and 50-day exposure of soil plots to Cu concentrations ranging from 150 mg to 450 mg kg^-1^, Wyszkowska et al. (2010) observed an impact of Cu on various enzymes, from most Cu-sensitive to least Cu-sensitive: alkaline phosphatase, arylsulfatase, acid phosphatase and β-glucosidase. Here we observed a recovery at the end of the experiment for both denitrification and Pase activities (but not LAP and β-glu). This suggests that heterotrophic communities exposed to Cu adapted and became partially resilient despite a very limited decrease in Cu concentrations in the sediment. Rajapaksha et al. (2004) studied thymidine incorporation rates in Cu-contaminated soils and also observed microbial community recovery within a few weeks. Functional resilience is often observed in microbial communities, but the degree to which resilience is possible depends on the functions measured (Allison and Martiny, 2008; Azarbad et al., 2016). Nevertheless, and according to the ARISA analysis, this functional recovery was not due to a structural recovery of the bacterial community. The lack of relationship between functional and structural recovery of heterotrophic microbial communities following a Cu exposure has already been observed by Lambert et al. (2012) in periphytic biofilms. Moreover, we observed a significant decrease in microbial respiration under Cu exposure at d21. This inhibition could reflect a functional cost of adaptation and resilience (Azarbad et al., 2016). It also underlines the importance of considering the temporal dynamics of the ecotoxicological effects that can occur under chronic exposure.

An originality of the approach employed here is that we also studied the effects of mixtures of Cu and As on sediment microbial communities. The structural and functional response of sediment microbial communities simultaneously exposed to Cu and As was almost similar to that observed under Cu exposure alone. Since As alone had very limited and sporadic effects on the studied parameters, we cannot firmly conclude on whether there are interaction effects between these two metals. Note, however, that whatever the functional parameter considered, it always tended to decrease at d21 in the MIX treatment compared to the Cu treatment, suggesting a potential interaction at the end of the period studied (even if this trend was only statistically significant for denitrification, *nosZ* I, *nirK* and *nirS* gene abundances). Such a hypothesis could be supported by the results obtained from denitrification measurements which revealed a recovery at d21 under Cu exposure alone whereas effects lasted until the end of the study when As was combined with Cu. This may reveal an additive or a synergetic effect of these metals on this microbial function. To our knowledge, there is no published data addressing the interactive effects of Cu and As on sediment microbial communities.

In summary, sediment microbial communities were structurally and functionally impaired by the chronic exposure to environmental concentrations of Cu. Exposure to As had almost undetectable effects, but when the two metals were combined, certain functional responses showed an interaction between Cu and As toxicity toward heterotrophic communities. This experimental study also revealed temporal dynamics in functional response of sediment communities to chronic Cu exposure, alone or in mixture, with some functions being resilient (denitrification and Pase activity) and others being impacted throughout the experiment (β-glu and Pase activities) or only after several weeks of exposure (respiration). Further studies are needed to better understand the adaptation mechanisms involved at different microbial levels (from genes to community) and to evaluate the resulting functional costs.

Given the ecological role of benthic microbial communities, our findings suggest that the sediment contamination by Cu (alone or combined with other metals) is likely to affect certain biochemical processes, thus potentially impairing aquatic ecosystem functioning. As recently emphasized by Pesce et al. (2018), this kind of experimental study confirms the need to improve the ecotoxicological assessment of sediments in freshwater environments by further considering the relationships between contaminant exposure and structural and functional effects at community level.

## Author Contributions

EL, SP, and AD conceived and designed the study. AMA and CB performed the experiments and samplings. AMA, CB, EL, and AD analyzed the samples. AMA, EL, and SP drafted the manuscript and CB and AD critically revised the article. All co-authors analyzed and interpreted the data, and approved the final submitted version of the manuscript.

## Conflict of Interest Statement

The authors declare that the research was conducted in the absence of any commercial or financial relationships that could be construed as a potential conflict of interest.
