# Supplementary material for: Environmental Concentrations of Copper, Alone or in Mixture With Arsenic, Can Impact River Sediment Microbial Community Structure and Functions
Source: Front Microbiol. 2018 Aug 14;9:1852. doi: 10.3389/fmicb.2018.01852 (PMC6104476; doi:10.3389/fmicb.2018.01852)
Supplement: Supplementary file 1 [file Data_Sheet_1.DOCX]

Supplementary Material

**Environmental concentrations of copper, alone or in mixture with arsenic, can impact river sediment microbial community structure and functions.**

Ayanleh Mahamoud Ahmed¹^,^²^,^³, Emilie Lyautey², Chloé Bonnineau¹, Aymeric Dabrin¹, Stéphane Pesce¹^*^

^1^Irstea, UR RiverLy, Centre de Lyon-Villeurbanne, Villeurbanne, France
 ^2^CARRTEL, Univ. Savoie Mont Blanc, INRA, Chambéry, France
 ^3^Centre de Recherche, Université de Djibouti, Djibouti, Djibouti

Correspondence :

Dr. Stéphane Pesce

[stephane.pesce@irstea.fr](mailto:stephane.pesce@irstea.fr)

**Supplementary Tables**

**Supplementary Table 1:** experimental conditions used for the estimation of total and functional community sizes using real-time quantitative PCR (qPCR).

| Gene | Primer pair (reference) | Primer concentration | Master mix | Thermocycler | Thermal conditions from |
| --- | --- | --- | --- | --- | --- |
| Bacterial 16S rRNA | 341F and 515R (López-Gutiérrez et al., 2004) | 0.3 µM | Brilliant II SYBR® Green Low ROX QPCR Master Mix (Agilent) | MX3005P qPCR system (Agilent) | López-Gutiérrez et al., 2004 |
| *nir*S | Cd3aF and R3cd (Kandeler et al., 2006) | 1 µM | QuantiTect SYBR Green Master Mix (Qiagen) | StepOnePlus real-time PCR System (Applied Biosystem) | Throbäck et al., 2004 |
| *nir*K | 876F and 1040R for the nirK gene (Henry et al., 2004) | 1 µM | QuantiTect SYBR Green Master Mix (Qiagen) | StepOnePlus real-time PCR System (Applied Biosystem) | Kandeler et al., 2006 |
| *nos*Z clade I | nosZ1F and nosZ1R (Henry et al., 2006) | 1 µM | Brilliant II SYBR® Green Low ROX QPCR Master Mix (Agilent) | MX3005P qPCR system (Agilent) | Henry et al., 2004, 2006 |
| *nos*Z clade II | nosZ-II-F and nosZ-II-R (Jones et al., 2013) | 1 µM | Brilliant II SYBR® Green Low ROX QPCR Master Mix (Agilent) | MX3005P qPCR system (Agilent) | Jones et al. 2013 |

**Supplementary Table 2:** Concentrations of Cu and As in circulating water (µg L^-1^) at d0, d7, d14 and d21. The four treatments are: sediment without contamination (REF), sediment contaminated with Cu (Cu), sediment contaminated with As (As), and sediment contaminated with both As and Cu (MIX).

|  | As (µg L^-1^) | | | | Cu (µg L^-1^) | | | |
| --- | --- | --- | --- | --- | --- | --- | --- | --- |
|  | d0 | d7 | d14 | d21 | d0 | d7 | d14 | d21 |
| REF | 0.6 | 1.3 | 1.0 | 0.7 | 1.3 | 1.5 | 1.9 | 2.3 |
| As | 0.5 | 1.5 | 1.7 | 1.1 | 60.1 | 55.6 | 50.6 | 38.9 |
| Cu | 759.4 | 2829 | 1455 | 863 | 1.3 | 1.7 | 4.9 | 5.0 |
| MIX | 798.4 | 2340 | 1533 | 902 | 68.9 | 59.9 | 50.4 | 41.4 |

**Supplementary Figures**


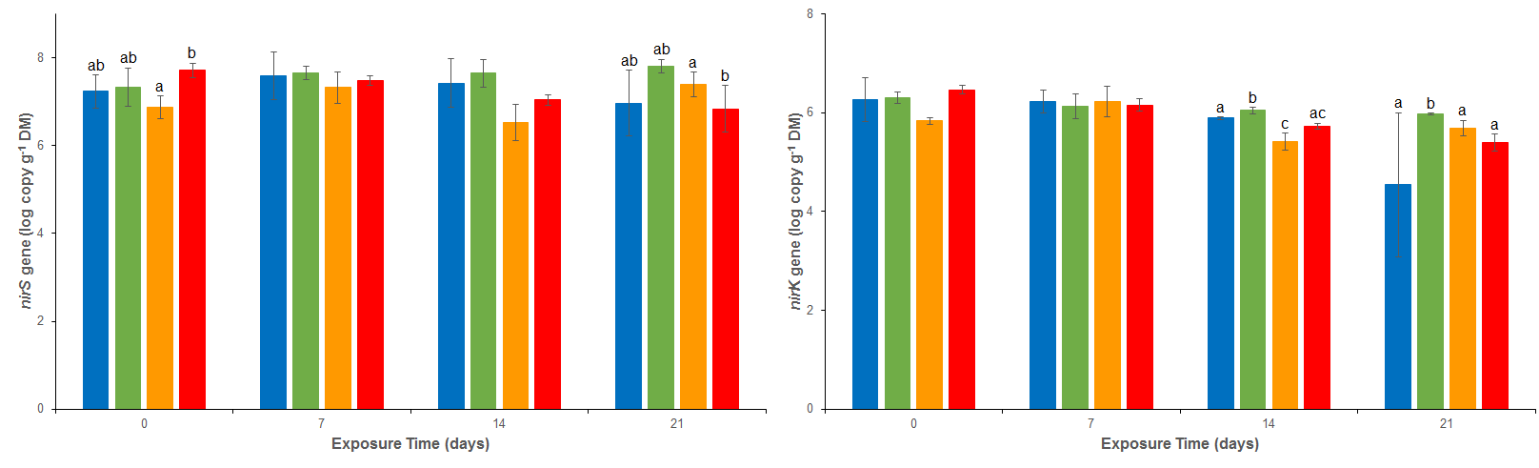


**Supplementary Figure 1:** Abundance of *nir*S (A) and *nir*K (B) genes analyzed by quantitative PCR in uncontaminated sediments (REF) and sediments contaminated with arsenic (As), copper (Cu) or As plus Cu mixture (MIX). For each sampling time, different letters indicate significant differences between treatments (*P*<0.05, Tukey test).


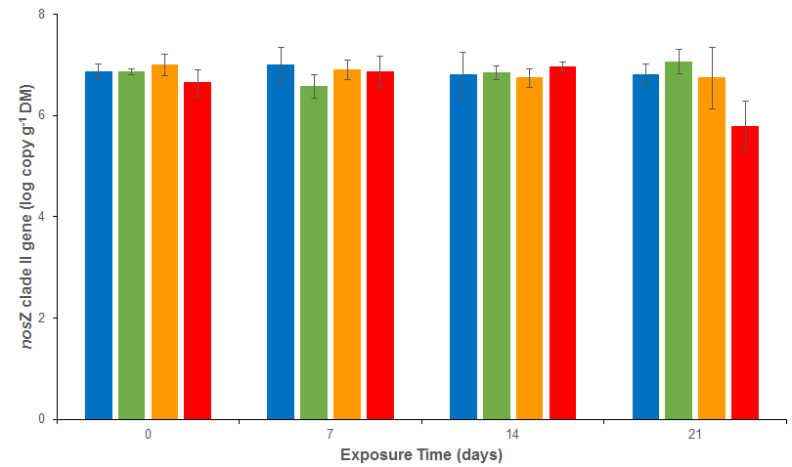


**Supplementary Figure 2:** Abundance of *nos*Z clade II genes analyzed by quantitative PCR in uncontaminated sediments (REF) and sediments contaminated with arsenic (As), copper (Cu), or As plus Cu mixture (MIX). No letter indicates the absence of significant differences between treatments (*P*<0.05, Tukey test).
